# Habitat Mosaic Limits Gene Flow and Promotes Morphological Adaptation in a Generalist Mammal

**DOI:** 10.1002/ece3.72588

**Published:** 2025-12-03

**Authors:** Warren Booth, W. Ian Montgomery, Lindsay S. Miles, Stephen H. Montgomery, Chris Harrod, Anja Schunke, Paulo A. Prodöhl

**Affiliations:** ^1^ Department of Entomology Virginia Polytechnic Institute and State University Blacksburg Virginia USA; ^2^ School of Biological Sciences Queen's University Belfast Belfast UK; ^3^ School of Biological Sciences University of Bristol Bristol UK; ^4^ Scottish Centre for Ecology and the Natural Environment, School of Biodiversity, One Health and Veterinary Medicine University of Glasgow Glasgow UK; ^5^ DogPersonality Plön Germany

**Keywords:** *Apodemus sylvaticus*, genetic differentiation, habitat specialization, habitat type, mandibular shape variation, stable isotope analysis

## Abstract

Many habitat generalist species exploit habitat patches of differing types and quality, yet the influence of such habitat mosaics on genetic structure remains poorly understood. Here, we tested whether fine‐scale habitat heterogeneity affects the population structure of the European wood mouse ( *Apodemus sylvaticus* ) by sampling three matched forest parks in Northern Ireland across hedgerow, forest edge, and inner forest habitats. Microsatellite analysis revealed strong genetic differentiation among sites and consistent divergence between habitat types within sites. Stable isotope data showed that hedgerow mice fed at a higher trophic level than inner forest individuals, with forest edge mice intermediate. Mandible shape also differed by habitat and was correlated with δ^15^N, though differences were subtle and may reflect both drift and plasticity. Together, these results indicate that habitat mosaics can promote repeated, fine‐scale population divergence even in the absence of physical barriers. This highlights the role of ecological heterogeneity in structuring genetic variation in widespread generalists and cautions against assuming panmixia in continuous landscapes.

## Graphical abstract

We investigated how fine‐scale habitat heterogeneity influences genetic structure in the European wood mouse by sampling individuals across hedgerow, forest edge, and inner forest habitats in three Northern Ireland forest parks. Microsatellite and stable isotope analyses revealed consistent genetic divergence and dietary differences among habitat types, with subtle but significant variation in mandible shape. These findings suggest that ecological mosaics can drive fine‐scale population divergence in habitat generalists, even without physical barriers.

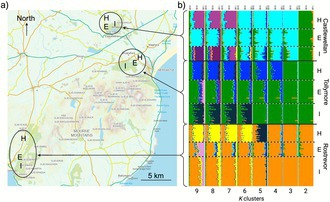

## 1 Introduction

Dispersal, defined as the movement of an individual from its birthplace to its reproductive site, is a key mechanism driving gene flow within and among populations (Matthysen 2012). However, dispersal is energetically costly and may increase predation risks, so individuals might prefer moving within familiar habitats rather than navigating diverse and complex habitat mosaics (Bowler and Benton 2005; Selonen et al. 2007). Individual dispersal biases significantly influence patterns of gene flow across heterogeneous landscapes (Balkenhol et al. 2015; Bustillo‐de la Rosa et al. 2024). Furthermore, habitat boundaries may shape genetic structure by acting as filters or barriers, allowing movement for some individuals while restricting others (Gortat et al. 2010; Holderegger et al. 2006; Tattersall et al. 2004). While certain barriers (e.g., rivers, roads, or habitat corridors) are readily identifiable, others may be less obvious. For instance, movement among distinct but contiguous habitat types may be constrained by an individual's preference for its natal habitat type (Davis and Stamps 2004; Stamps and Swaisgood 2007). Studies on various species show that dispersers often choose habitats like their birthplace, even when alternatives are available (Hooven et al. 2023; Mabry and Stamps 2008; Orgeret et al. 2024; Piper et al. 2013). Despite growing evidence that habitat type influences dispersal (Hannebaum et al. 2017; Haughland and Larsen 2004; Mabry and Stamps 2008; Selonen et al. 2007), studies addressing its effect on population genetic structure are limited (Baptista et al. 2021; Johnson et al. 2023; Maritinez et al. 2018). This research gap is particularly notable for widespread, highly vagile, habitat generalists, in which marked population genetic structure might not be expected (Cancellare et al. 2021; Szulkin et al. 2016; Porlier et al. 2012).

Distinct habitat types may impose varying selection pressures on generalist species within mosaic landscapes. These pressures can drive phenotypic trait evolution through either non‐adaptive divergence via genetic drift or adaptive evolution in response to habitat‐specific conditions (Ravinet et al. 2013; Schroeder et al. 2022). Adaptive trait development in ecologically distinct habitats may lead to divergent selection, creating gene flow barriers between populations and resulting in isolation‐by‐adaptation (Nosil et al. 2008), potentially enhancing population divergence through genetic drift. The extent and distribution of trait variation within and among populations remain poorly understood (Boell and Tautz 2011), despite their importance for interpreting micro‐evolutionary patterns.

One trait receiving significant attention is mandibular morphology, as environmental factors such as diet and trophic level strongly influence its shape and function (Boell and Tautz 2011; Brum et al. 2022; Maestri et al. 2016; Martínez et al. 2025; Renaud and Auffray 2009; Renaud and Michaux 2003; von Cramon‐Taubadel 2011). Previous studies examined mandible differentiation at broad geographic scales, ranging from hundreds to thousands of kilometers (Renaud 2005; Renaud and Auffray 2009; Renaud and Michaux 2003). However, fine‐scale geographic variation remains largely unexplored, despite the potential for localized genetic structure and habitat‐driven differentiation (Booth et al. 2009; Ravinet et al. 2013; Rudyk et al. 2025). The presence of fine‐scale population genetic structure may also confound interpretations of studies at landscape or geographical scales.

Phenotypic differences in the masticatory apparatus have been documented in wood mice, *Apodemus sylvaticus* (Renaud 2005), a habitat generalist with considerable dispersal potential (O'Neill 2001; Wolton 1985; Wolton and Flowerdew 1985) yet notably strong population structure (Berkmoes et al. 2005; Booth et al. 2009; Wilson et al. 2016). Although primarily granivorous, resource availability across habitats may shift the diet toward animal or plant material (Montgomery and Montgomery 1990; Rogers and Gorman 1995). However, it is unclear whether these variations result from local evolutionary processes or simply reflect intra‐population phenotypic plasticity (Renaud 2005; Renaud and Michaux 2003). Extensive ecological and behavioral data for *A. sylvaticus* (Harris and Yalden 2008; Montgomery and Gurnell 1985), combined with population genetic, morphometric, and trophic‐level analyses, provide an opportunity to test whether adjacent yet ecologically distinct habitats, lacking physical dispersal barriers, affect genetic population subdivision. This study employs replicated analyses across temporal and spatial scales to investigate putative evolutionary outcomes through an eco‐morphological lens.

## 2 Methods

### 2.1 Survey Design and Study Sites

**FIGURE 1:**
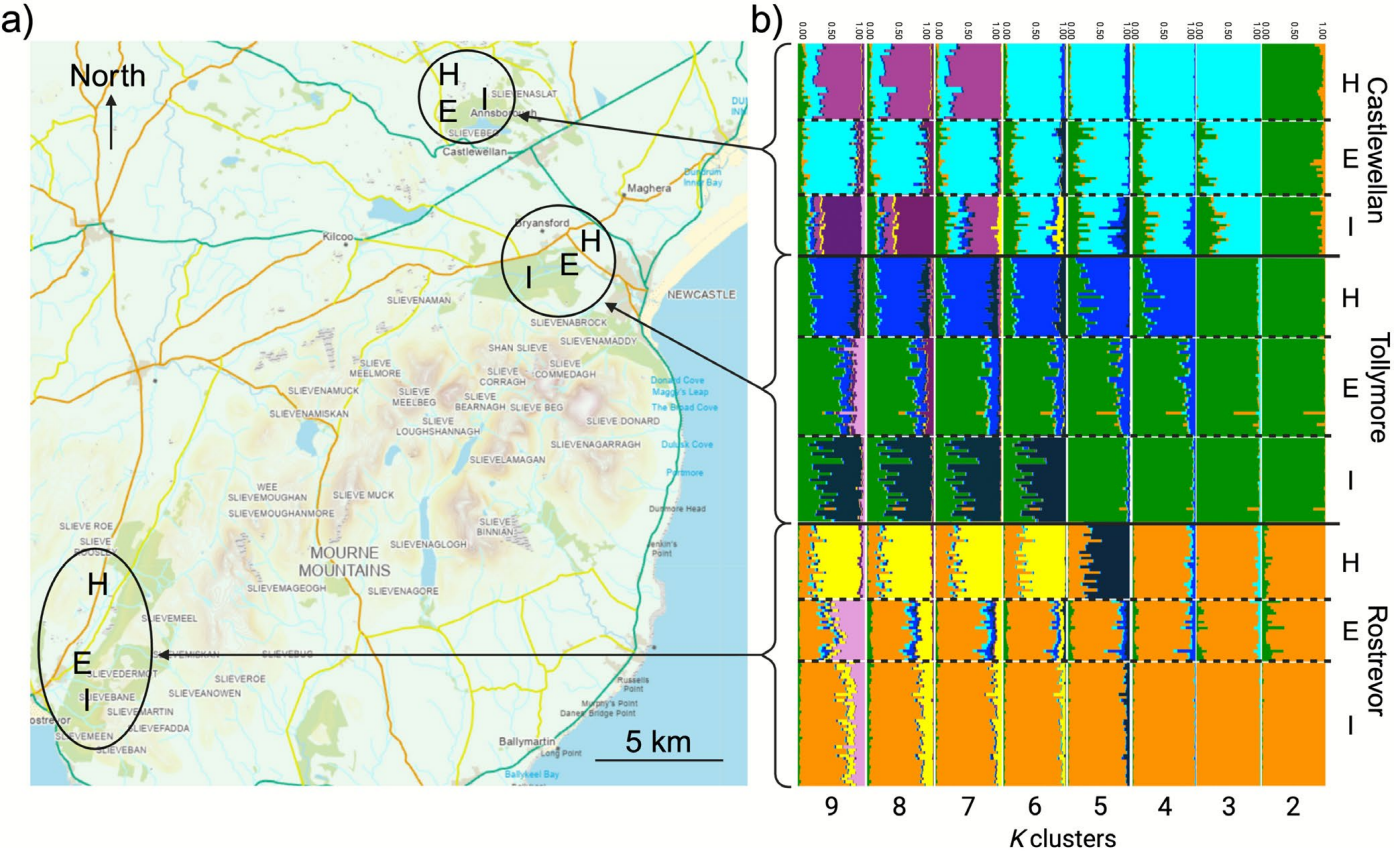
(a) Map of Mourne Mountains and surrounding area showing the three study sites, Castlewellan (top), Tollymore (middle), and Rostrevor (bottom) Forest Parks (dark green) and adjacent farmland (light green). Three habitats, hedgerow (H), forest edge (E), and inner forest (I) are indicated for each study site. Ordnance Survey of Northern Ireland Spatial NI Map Viewer. Dept of Agriculture, Environment and Rural Affairs. (b) Summary of Bayesian clustering analysis (STRUCTURE, Pritchard et al. 2000) for wood mice ( *Apodemus sylvaticus* ) sampled from three forest parks in Northern Ireland. Individuals were collected from hedgerows (H), forest edges (E), and inner forests (I) in Tollymore (T), Castlewellan (C), and Rostrevor (R), with site–habitat codes shown as TH, TE, TI, CH, CE, CI, RH, RE, and RI. Each bar represents one individual, partitioned into genetic clusters according to estimated membership coefficients. Analyses were performed across *K* = 2–9, with results summarized using CLUMPAK. The figure illustrates clear subdivision among forest parks and, within parks, among habitat types, providing strong evidence that both spatial and ecological boundaries contribute to fine‐scale genetic structure.

This study was conducted at three matched sites: Tollymore (Irish grid ref. J345320), Castlewellan (J330375), and Rostrevor (J195175) Forest Parks in County Down, Northern Ireland. At each site, samples were collected from three distinct habitat types: hedgerows in pastoral farmland, forest edges, and inner forests, with habitat patches separated by 1.0–2.5 km (Figure 1a). Distances were chosen according to the species' documented dispersal ability (O'Neill 2001; Wolton 1985; Wolton and Flowerdew 1985). Hedgerows were dominated by Hawthorn ( *Crataegus monogyna* ), Blackthorn ( *Prunus spinosa* ), Hazel ( *Corylus avellana* ), and Holly ( *Ilex aquifolium* ); forest edges by Ash ( *Fraxinus excelsior* ), Beech ( *Fagus sylvatica* ), Douglas fir (*Pseudotsuga menziesii*), Norway spruce ( *Picea abies* ), European larch (*Larix decidua*), and Japanese larch ( *L. kaempferi* ); and inner forests by Oaks spp. ( *Quercus robur* and *Q. petraea* ), Beech, Norway spruce, and European larch. The basic landscape pattern of forest bounded by farmland with hedgerows dates from at least the eighteenth century at Tollymore and Castlewellan, originally family‐run estates, whereas Rostrevor forest was developed around remnant regenerated oak woodland, last felled around 250 years ago (House of Commons Report 1971).

Sampling at Tollymore Forest occurred in January 2004 and 2005, while Castlewellan and Rostrevor were sampled in January 2005. In 2004, trap‐lines consisted of 25 points spaced 10 m apart, each with two Longworth live traps. In 2005, these were replaced by Self‐set snap traps. Each site was trapped for three consecutive nights, with traps checked daily between 7:00 a.m. and 10:00 a.m. For stable isotope and mandible morphology analysis, mice were captured using Self‐set break back traps during February 2008 at the same locations. Trapping was conducted with permission from the Forest Park Service of Northern Ireland and complied with Queen's University Belfast's animal handling protocols and relevant legal guidelines. *Apodemus sylvaticus* is a common rodent species and not subject to conservation legislation.

### 2.2 Population Genetic Analysis

Tail tissue biopsies (1 cm) were collected from each specimen and preserved in 99% molecular‐grade ethanol at −20°C. DNA extraction followed the phenol‐chloroform method described by Taggart et al. (1992). Individuals were screened at seven polymorphic microsatellite loci, as follows: As‐7, As‐20, As‐34 (Harr et al. 2000); GCATD7S, TNF(CA) (Makova et al. 1998); MSAF‐8 (Gockel et al. 1997); and WM2 (Barker 2002). PCR conditions for analysis on a LiCor 4300 dual laser DNA analyzer (Lincoln, Nebraska, USA) followed Booth et al. (2007).

Genetic diversity was assessed as observed (*H*_O_) expected heterozygosity (*H*_E_) using the R package *diveRsity* (Keenan et al. 2013). Allelic richness (*A*r) was estimated using rarefaction in HP‐RARE (Kalinowski 2005). To reduce bias caused by small sample sizes (*n* < 30) (Leberg 2002), analyses were standardized to a common sample size of 21. Deviations from Hardy–Weinberg equilibrium (HWE) were tested in GENEPOP using exact probability tests with Markov chain parameters of 10,000 dememorizations, 100 batches, and 1000 iterations per batch. Significance levels were adjusted for multiple comparisons using sequential Bonferroni correction. Weir and Cockerham (1984) estimate of *F*_ST_ was calculated both pairwise and among populations using *diveRsity* (Keenan et al. 2013). To account for potential sex‐biased dispersal (O'Neill 2001), tests were carried out for individual sexes separately.

Population structure was examined using STRUCTURE 2.3.4 (Pritchard et al. 2000) and discriminant analyses of principal components (DAPC) (Jombart et al. 2010). STRUCTURE analyses employed the admixture model with correlated allele frequencies, using a burn‐in of 100,000 followed by 100,000 MCMC iterations, with 20 replicates for each *K* (2–15). To address both broad and fine‐scale structure, analysis was performed without and with the use of a ‘population prior’, respectively. The latter provides external information (i.e., sampling location) to aid the model in grouping individuals and shaping genetic structure inference. The most likely *K* was inferred using the Δ*K* method (Evanno et al. 2005), and results were summarized with CLUMPAK (Kopelman et al. 2015). DAPC was implemented in adegenet (v2.1.11) (Jombart and Ahmed 2011). The optimal *K* was determined via BIC. The number of principal components retained (45) was identified using cross‐validation, and two discriminant functions were used to describe differentiation. Given the lack of significant genetic differentiation between Tollymore temporal samples (see Section 3: Results), DAPC was restricted to samples collected in 2005.

### 2.3 Trophic Ecology

Muscle tissue was dissected from the rear leg of each snap‐trapped individual captured in 2008. Samples were dried at 60°C for 24 h and ground to a fine powder in an agate mortar and pestle. Approximately 0.5 mg of each sample was weighed into tin cups and combusted in a Eurovector elemental analyzer (Eurovector, Milan, Italy) coupled to a Micromass Isoprime continuous‐flow isotope ratio mass spectrometer (Micromass, Manchester, UK) at the Max Planck Institute for Evolutionary Biology, Plön, Germany. Stable isotope ratios are reported in δ notation expressed in units of per mil (‰), using the equation: *δ* (‰) = [(*R*_sample_/*R*_standard_) − 1] × 1000, where *R* = ^13^C/^12^C or ^15^N/^14^N in the sample (*R*_sample_) or reference standard (*R*_standard_). Reference materials were calibrated against international standards: Vienna Peedee Belemnite (carbon) and atmospheric N_2_ (nitrogen). Analytical precision, based on repeated measurements of internal standards (one after every six samples) was < 0.1‰ for δ^13^C and < 0.3‰ for δ^15^N.

Mean δ^13^C and δ^15^N values were analyzed using two‐way ANOVAs with habitat, forest, and their interactions as fixed effects. Significant interaction terms (forest × habitat, Tables S1 and S2), prompted subsequent within‐forest comparisons using one‐way ANOVAs. Given restricted sample sizes and unbalanced designs, a conservative significance threshold of *p* = 0.01 was applied for ANOVAs and Tukey's HSD post hoc tests.

### 2.4 Mandibular Morphometric Analysis

Mandible images were obtained from thawed whole specimens using a vivaCT 40 microCT scanner (Scanco Medical AG), which generated virtual radiographs of lateral views from hemimandibles (Figure S1). Morphometric data were obtained from 87 specimens with at least one intact hemimandible (Table S2). Two data sets were analyzed: one based on 15 standard landmarks following Klingenberg et al. (2004) (landmarks 1–7 and 9–16), and another including 10 additional landmarks (tip of the incisor; ventral and dorsal lowest point of the incisor within the mandible bone; posterior end of the incisor alveolus; anterior tip of the first molar tooth; anterior tip of the first alveolus; posterior tip of the third molar; posterior tip of the last alveolus; ventral‐most point of the mandible between coronoid and articular process; posterior end of the articular surface; Figure S1). Landmarks were digitized using tpsDig2 (Rohlf 2006), and statistical analyses (principal component analysis [PCA] and canonical variate analysis [CVA]) were conducted with CoordGen6f, PCAGen6n, and CVAGen6k from the IMP software suite (Sheets 2003).

## 3 Results

### 3.1 Population Genetic Differentiation

A total of 350 specimens of wood mouse from three habitat types across three forest parks were successfully genotyped at seven microsatellite loci (Table 1). All loci amplified reliably and could be scored without ambiguity. Across loci, allelic diversity was moderate to high, ranging from 2 (WM2; Tollymore 2005, inner forest) to 23 alleles (MSAF‐8; Tollymore 2004, forest edge), with a mean of 11.0 alleles per locus per sampling location. When averaged across loci, allele richness ranged from 9.0 (Tollymore 2005, hedgerow) to 10.9 (Tollymore 2004, inner forest). Observed heterozygosity was generally high, averaging 0.74 across all samples, and ranged from 0.67 (Tollymore 2005, inner forest) to 0.79 (Castlewellan 2004, hedgerow; Rostrevor 2005, inner forest). Expected heterozygosity showed similar patterns (mean = 0.78). Departures from Hardy–Weinberg equilibrium were infrequent: only Castlewellan hedgerow (GCATD7S, *p* < 0.001), Tollymore inner forest 2005 (As‐20, *p* < 0.001), and Rostrevor forest edge (TNF(CA), *p* < 0.001) exhibited significant deviations after Bonferroni correction. No single habitat type consistently exhibited greater or reduced allelic richness or heterozygosity, suggesting broadly comparable levels of genetic diversity across hedgerow, forest edge, and inner forest environments (Table 1).

**TABLE 1:** Summary statistics for the wood mouse, *Apodemus sylvaticus* , samples screened for seven microsatellite loci. All loci amplified consistently, and genotypes could be unambiguously scored in every case.

Sampled location	Summary statistics	Microsatellite loci							
		*As‐*7	*As‐*20	*As‐*34	GCATD7S	MSAF‐8	WM2	TNF(CA)	Avg
Tollymore Hedgerow 2004 (*N* = 27)	*A*	12	11	11	8	16	4	10	10.3
	*Ar*	11.4	10.6	10.4	7.3	15.4	3.9	9.3	9.8
	*H*_O_	0.93	0.63	0.93	0.74	0.81	0.52	0.89	0.78
	*H*_E_	0.88	0.88	0.85	0.75	0.92	0.48	0.83	0.81
	HWE	ns	ns	ns	ns	ns	ns	ns	ns
Tollymore Edge 2004 (*N* = 46)	*A*	12	17	11	9	23	4	12	12.6
	*Ar*	9.9	13.7	8.9	7.4	18.4	3.4	10.4	10.3
	*H*_O_	0.65	0.85	0.67	0.78	0.8	0.26	0.85	0.7
	*H*_E_	0.81	0.91	0.75	0.73	0.96	0.31	0.86	0.76
	HWE	ns	ns	ns	ns	ns	ns	ns	ns
Tollymore Inner 2004 (*N* = 22)	*A*	12	12	12	10	16	3	12	11
	*Ar*	11.8	12.0	11.9	9.9	16.0	3.0	11.9	10.9
	*H*_O_	0.77	0.77	0.91	0.77	0.77	0.18	0.82	0.71
	*H*_E_	0.88	0.89	0.86	0.76	0.92	0.55	0.88	0.79
	HWE	ns	ns	ns	ns	ns	ns	ns	ns
Tollymore Hedgerow 2005 (*N* = 34)	*A*	11	11	10	6	14	5	10	9.6
	*Ar*	10.6	10.2	9.1	5.9	13.4	4.6	9.6	9.0
	*H*_O_	0.96	0.96	0.79	0.61	0.68	0.25	0.82	0.72
	*H*_E_	0.86	0.86	0.77	0.65	0.89	0.34	0.84	0.74
	HWE	ns	ns	ns	ns	ns	ns	ns	ns
Tollymore Edge 2005 (*N* = 28)	*A*	8	14	10	8	21	3	10	10.6
	*Ar*	7.7	12.4	8.6	7.4	18.4	2.9	9.8	9.6
	*H*_O_	0.74	0.97	0.68	0.82	0.68	0.18	0.85	0.7
	*H*_E_	0.73	0.89	0.75	0.79	0.92	0.42	0.88	0.75
	HWE	ns	ns	ns	ns	ns	ns	ns	ns
Tollymore Inner Forest 2005 (*N* = 30)	*A*	11	13	12	9	13	2	11	10.1
	*Ar*	10.5	12.0	10.4	8.3	12.9	2.0	10.6	9.5
	*H*_O_	0.67	0.9	0.63	0.8	0.77	0.1	0.83	0.67
	*H*_E_	0.86	0.89	0.86	0.79	0.92	0.21	0.87	0.77
	HWE	ns	**< 0.001**	ns	ns	ns	ns	ns	ns
Castlewellan Hedgerow 2004 (*N* = 26)	*A*	9	12	11	8	17	5	11	10.4
	*Ar*	8.8	11.5	10.7	7.7	16.4	4.8	10.7	10.1
	*H*_O_	0.81	0.92	0.85	0.73	0.88	0.46	0.85	0.79
	*H*_E_	0.82	0.86	0.87	0.74	0.92	0.59	0.88	0.82
	HWE	ns	ns	ns	**< 0.001**	ns	ns	ns	ns
Castlewellan Edge (*N* = 26)	*A*	10	12	8	10	16	3	9	9.7
	*Ar*	9.6	11.5	7.8	9.5	15.0	3.9	8.4	9.4
	*H*_O_	0.77	0.88	0.77	0.65	0.85	0.12	0.81	0.69
	*H*_E_	0.84	0.84	0.83	0.85	0.81	0.41	0.8	0.76
	HWE	ns	ns	ns	ns	ns	ns	ns	ns
Castlewellan Inner Forest (*N* = 21)	*A*	12	13	10	7	17	4	12	10.7
	*Ar*	12.0	13.0	10.0	7.0	17.0	4.0	12.0	10.7
	*H*_O_	0.76	0.95	0.67	0.76	0.9	0.33	0.81	0.74
	*H*_E_	0.81	0.87	0.82	0.8	0.92	0.52	0.88	0.8
	HWE	ns	ns	ns	ns	ns	ns	ns	ns
Rostrevor Hedgerow (*N* = 21)	*A*	15	13	9	8	18	7	11	11.6
	*Ar*	13.8	12.2	8.8	7.7	16.3	6.1	10.6	10.8
	*H*_O_	0.96	0.96	0.59	0.7	0.93	0.41	0.67	0.75
	*H*_E_	0.89	0.88	0.83	0.74	0.91	0.35	0.85	0.78
	HWE	ns	ns	ns	ns	ns	ns	ns	ns
Rostrevor Edge (*N* = 27)	*A*	12	12	8	7	13	4	11	9.6
	*Ar*	12.0	12.0	8.0	7.0	13.0	4.0	11.0	9.6
	*H*_O_	0.71	0.76	0.71	0.71	0.81	0.24	0.9	0.69
	*H*_E_	0.89	0.87	0.82	0.77	0.88	0.22	0.82	0.77
	HWE	ns	ns	ns	ns	ns	ns	**< 0.001**	ns
Rostrevor Inner Forest (*N* = 43)	*A*	13	13	12	8	13	6	12	11
	*Ar*	10.9	11.8	9.8	6.7	11.0	4.8	9.9	9.3
	*H*_O_	0.86	0.88	0.84	0.74	0.81	0.6	0.79	0.79
	*H*_E_	0.83	0.89	0.86	0.78	0.84	0.54	0.83	0.8
	HWE	ns	ns	ns	ns	ns	ns	ns	ns

*Note:* Significant tests following Bonferroni correction are displayed in bold; Avg = average value for *A, H*_o_ and *H*_e_, and combined value for HWE over the seven loci.

Abbreviations: *A* = number of alleles, *Ar* = allele richness, *H*_o_ = observed heterozygosity, *H*_e_ = expected heterozygosity, HWE = exact tests for non‐conformance to Hardy–Weinberg Expectations, *N* = number of individuals screened per sample, ns = nonsignificant.

**FIGURE 2:**
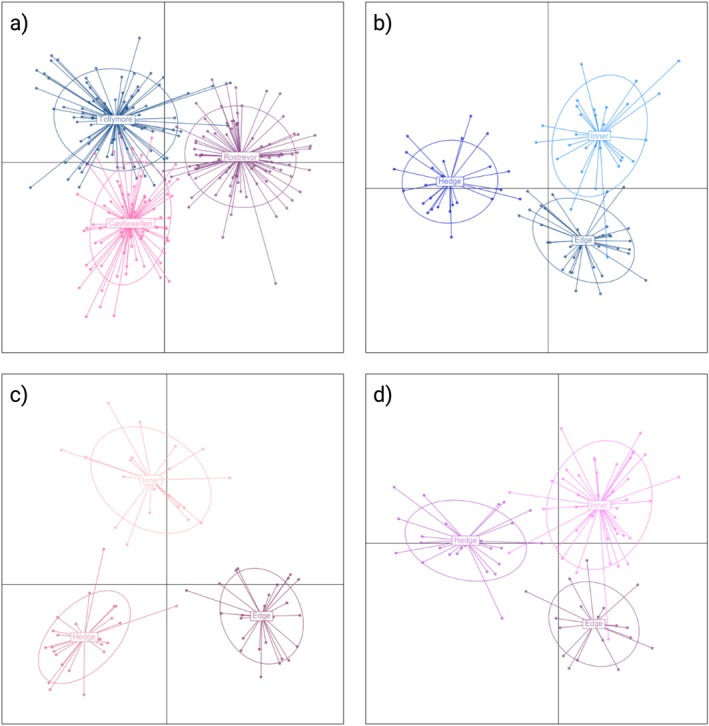
Discriminant analysis of principal components (DAPC) scatter plots of wood mice ( *Apodemus sylvaticus* ) sampled in 2005 from three forest parks in Northern Ireland (Tollymore, Castlewellan, and Rostrevor). Panel (a) shows all three parks combined, while panels (b–d) show each park separately—b = Tollymore, c = Castlewellan, d = Rostrevor. The plots illustrate strong genetic separation among forest parks and clear differentiation among habitat types within each park.

STRUCTURE analysis revealed clear genetic subdivision across the data set, with distinct clusters separating the three forest parks identified without the addition of “population priors” (best *K* was between 2 and 3), and, importantly, consistent differentiation among habitat types within parks when “population priors” were applied (best *K* was between 8 and 9) (Figure 1b). Please note that finding the “best” number of populations (*K*) is often seen as the main goal of clustering analyses, but this can be misleading. Programs including STRUCTURE create simplified models of genetic variation that do not always reflect real biological populations. Instead, these models are tools that help us summarize complex patterns in the data. As others have noted (Jombart et al. 2010; Meirmans 2015), there is no single “true” *K*. Different values of *K* can simply provide useful summaries of the data. For this reason, methods such as the Evanno et al. (2005) approach should be viewed as guides rather than final answers, and their results should be interpreted together with ecological context, geography, and other analyses. STRUCTURE results were independently corroborated by DAPC, which also recovered strong separation among parks (Figure 2a), and clear partitioning of samples by habitat within each park (Figure 2b–d). The concordance between STRUCTURE and DAPC provides robust evidence that both forest park boundaries and habitat type act as barriers to gene flow at fine spatial scales.

Pairwise *F*_ST_ estimates (Table S1) mirrored the clustering results. Across all comparisons, *F*_ST_ ranged from 0.004 to 0.064 (mean ≈ 0.033), consistent with the overall multilocus *F*_ST_ = 0.0339 (95% CI: 0.0277–0.0410). The strongest divergence occurred between Rostrevor and the other sites (up to *F*_ST_ ≈ 0.064, 95% CI: 0.042–0.087), while differentiation among habitats within parks was lower but significant (e.g., Tollymore 2004: *F*_ST_ = 0.020, CI: 0.010–0.036; Tollymore 2005: 0.029, 0.019–0.040; Castlewellan 2005: 0.032, 0.021–0.046; Rostrevor 2005: 0.030, 0.010–0.030; Table S1). Temporal comparisons at Tollymore revealed that differentiation between years for the same habitat (e.g., TH04 vs. TH05 ≈ 0.012) were non‐significant, suggesting stability of allele frequencies (Table S1 and Figure S2). No evidence was found to support sex‐biased differences in *F*_ST_ comparisons (Figure S3).

### 3.2 Trophic Ecology

**FIGURE 3:**
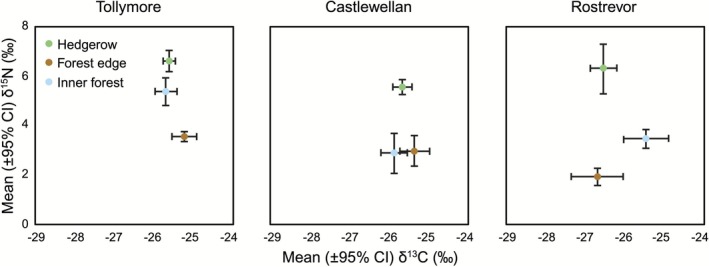
Variation in muscle δ^13^C and δ^15^N of wood mice ( *Apodemus sylvaticus* ) collected in 2008 from hedgerow, forest edge, and inner forest habitats in three forest parks (Tollymore, Castlewellan, Rostrevor), Northern Ireland.

Stable isotope analysis (δ^13^C and δ^15^N) revealed consistent differences in the trophic ecology of wood mice across habitat types. Mean δ^13^C values varied by less than 1.2‰ between habitats within forest parks (Figure 3, Table 2). In contrast, within each site, individuals from hedgerows exhibited significantly elevated δ^15^N values, typically enriched by more than 3.0‰ relative to those from inner forest habitats (Figure 3, Table 2). At two sites (Rostrevor and Tollymore), forest edge mice showed intermediate δ^15^N values, falling between those recorded for hedgerow and inner forest individuals.

**TABLE 2:** Mean δ^13^C and δ^15^N values (±SD) of wood mice ( *Apodemus sylvaticus* ) collected in 2008 from hedgerow, forest edge, and inner forest habitats in three forest parks (Tollymore, Castlewellan, Rostrevor), Northern Ireland.

Site	Habitat	Mean δ^13^C (±SD)	Mean δ^15^N (±SD)	*n*
Tollymore	Hedgerow	−25.2 (±0.23)^nsd^	6.6 (±0.64)^C^	18
	Forest‐edge	−25.7 (±0.61)^nsd^	5.3 (±1.17)^B^	20
	Inner forest	−25.6 (±0.63)^nsd^	3.5 (±0.44)^A^	11
	ANOVA	*F*_2,46_ = 3.82, *p* = 0.03	*F*_2,46_ = 47.1, *p* < 0.0001	
Castlewellan	Hedgerow	−25.7 (±0.31)^nsd^	6.9 (±0.51)^J^	9
	Forest‐edge	−25.9 (±0.5)^nsd^	3.6 (±1.52)^I^	11
	Inner forest	−25.4 (±0.44)^nsd^	3.7 (±0.9)^I^	8
	ANOVA	F_2,25_ = 3.59, *p* = 0.04	F_2,25_ = 26.2, *p* < 0.0001	
Rostrevor	Hedgerow	−26.5 (±0.47)^e^	6.3 (±1.37)^G^	10
	Forest‐edge	−25.5 (±0.68)^f^	3.4 (±0.47)^F^	8
	Inner forest	−26.7 (±0.7)^e^	1.9 (±0.4)^E^	7
	ANOVA	F_2,22_ = 9.68, *p* = 0.001	F_2,22_ = 48.1, *p* < 0.0001	

*Note:* ANOVA results and Tukey HSD post hoc comparisons are shown. Superscripts (lower case for δ^13^C, upper case for δ^15^N) indicate groups not significantly different at *α* = 0.01. Sample sizes (*n*) are given for each habitat × site combination.

### 3.3 Mandibular Morphometric Analysis

**FIGURE 4:**
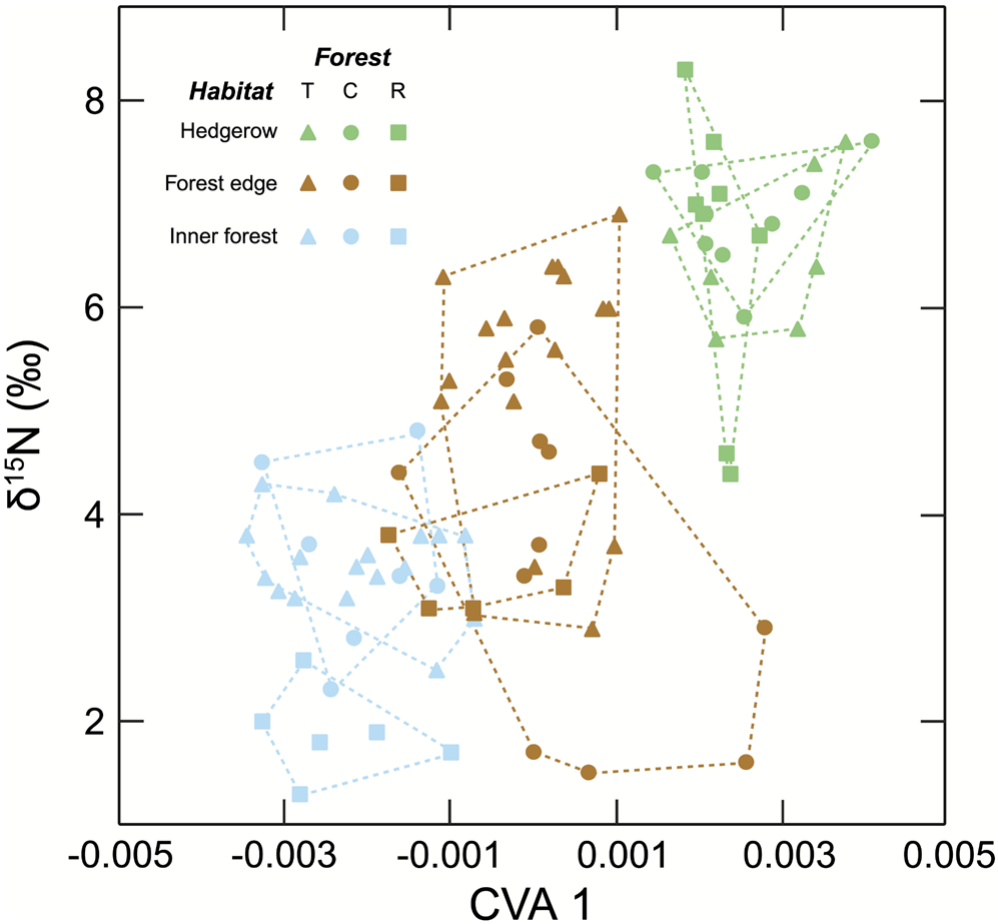
Relationship between mandible shape and trophic level (δ^15^N) in wood mice ( *Apodemus sylvaticus* ) collected in 2008 from hedgerow, forest edge, and inner forest habitats in three forest parks (Tollymore, Castlewellan, Rostrevor), Northern Ireland. Mandible shape variation is shown along CVA1 based on 25 landmarks. Polygons represent convex hulls including all individuals from each forest–habitat combination.

Principal components analyses did not distinguish specimens by forest or habitat type (results not shown). In contrast, canonical variates analysis (CVA) based on 25 landmarks revealed clear separation among habitat types (Wilks' lambda = 0.073, df = 92, *p* < 0.0001; Figure 4) and weaker, though significant, separation among forests (Wilks' lambda = 0.1243, df = 92, *p* = 0.008; results not shown). PCA outcomes were similar for both landmark sets, whereas CVA yielded stronger discrimination when the full set of 25 landmarks was used. Mandible shape and δ^15^N were correlated (*r* = 0.66, *n* = 87, *p* < 0.0001; Figure 4). Inner forest individuals clustered at lower trophic levels with distinct mandibular morphology, while hedgerow individuals showed high trophic levels and contrasting morphology. Forest edge individuals were intermediate in both morphology and diet. Visual examination of deformation grids based on variation along the first CV axis indicated that the shape of the jaws of individuals captured from Hedgerow habitats were relatively slim, contrasting with the more robust jaw shape of individuals from the Inner forest. Our findings align with previous research showing that rodents feeding primarily on seeds and other plant materials tend to possess more robust jaw structures, reflecting adaptations for generating higher bite forces (Samuels 2009).

## 4 Discussion

*Apodemus sylvaticus* is a geographically widespread species occupying a broad range of habitat types throughout Europe. Despite this, genetic structure has been detected previously at micro‐ and macrogeographic scales, both among natural sites and those within urban environments (Biello et al. 2022; Booth et al. 2009; Wilson et al. 2016). This suggests that while they have the ability and propensity to move over large distances relative to their size—radiotelemetry studies have revealed movement in excess of 400 m per night and over 1 km for longer durations (O'Neill 2001; Tattersall et al. 2004; Wolton 1985; Wolton and Flowerdew 1985)—such dispersal does not translate into gene flow, hinting at the existence of prezygotic barriers to reproduction, such as isolation driven by habitat type (Feder et al. 2012; Kozakiewicz and Jurasińska 1989; Nosil et al. 2007). Our results confirm and extend these earlier findings. STRUCTURE and DAPC both revealed strong subdivision among habitat types within forest parks, and pairwise *F*_ST_ estimates corroborated this pattern. While restricted to a single site, mitochondrial PCR‐RFLP analysis further supports population subdivision based on habitat type, with each habitat type exhibiting a distinct composite haplotype (see Supporting Information: Figure S4). Temporal comparisons between 2004 and 2005 habitat‐matched pairs at Tollymore showed non‐significant differentiation (Figure S2), demonstrating short‐term stability, while comparisons with earlier surveys (1979 and 2002) support long‐term stability (Booth 2005; Booth et al. 2009). In addition, no evidence of sex‐biased dispersal was detected (Figure S2), suggesting that both males and females contribute equally to restricted gene flow. Based on a combination of nuclear microsatellite markers, mitochondrial PCR‐RFLP, stable isotope, and mandibular morphology, our results show that *A. sylvaticus* is genetically structured even across proximate spatial scales, and that individuals may limit their movement and reproduction to natal habitat types.

Our findings also support previous studies detecting broad‐scale genetic structure in *A. sylvaticus* . As might be expected, regardless of habitat type, sites located nearby (Tollymore and Castlewellan ~5 km) exhibited greater genetic similarity than sites farther apart (Rostrevor, located ~25 km from both Tollymore and Castlewellan; Figures 1b, 2a). This is likely driven by isolation‐by‐distance (Booth et al. 2009), accentuated by the physical barrier of the Mourne Mountains, a granite mountain range largely inhospitable to small mammal movement outside of the forests and agricultural areas located in the foothills. The consensus across STRUCTURE, DAPC, and *F*_ST_ provides robust evidence that geographic distance restricts gene flow. Patterns of genetic divergence observed within and among sites also suggest that subdivision between habitat types arose independently at each of the three forest parks, pointing to a repeated and intimate relationship between landscape mosaic and genetic structure. Given that the present habitat mosaic has been established for up to 350 mouse generations (assuming mice may breed in the year of their birth 1 year out of two), there has been ample evolutionary time for such independent divergence to develop and persist.

The significant structuring detected between forest edge and inner forest habitats at all three sites was unexpected. *Apodemus sylvaticus* is often considered an ecotonic, soft‐edge species whose distribution is strongly affected by edge effects in continental Europe (Garcia et al. 1998; Telleria et al. 1991). This edge tendency has not been documented in the British Isles, where potential competitors are fewer in comparison to mainland Europe. Our finding that edge populations show genetic, morphological, and trophic differentiation suggests that British wood mice may retain an evolutionary legacy of edge specialism, with less competitive individuals displaced into inner forests.

Numerous ecological and behavioral factors may shape genetic patterns across habitat types (McCracken and Bradbury 1981; Singleton and Hay 1983). One such factor in *A. sylvaticus* is diet (Montgomery and Montgomery 1990). Thus, we hypothesized that the genetic substructuring observed here reflects local adaptation to foods available in each habitat. Stable isotope analysis (δ^13^C and δ^15^N) showed marked and repeated differences in the trophic ecology among habitats. Variation in mean δ^13^C values (< 1.2% between habitats; Figure 3 and Table 2) suggests broadly similar basal energy sources. Hedgerow mice were consistently enriched by > 3‰ in δ^15^N relative to inner forest individuals, a difference equivalent to approximately one trophic level and ecologically meaningful, given that fractionation between small rodents and their food is typically ~3% (DeNiro and Epstein 1981; Minagawa and Wada 1984). At Rostrevor and Tollymore, forest edge mice had δ^15^N values intermediate between those of hedgerow and inner forest individuals, consistent with a more omnivorous diet, whereas at Castlewellan they more closely resembled inner forest individuals. Nonetheless, δ^15^N values varied considerably among individuals regardless of site or habitat (Figure 4).

Given the marked dietary differences among habitats, we expected associated morphological divergence in the masticatory apparatus, as proposed by Renaud (2005), who suggested that mandible morphology may be under local selective or functional constraints. The mandible is a well‐established model for comparative analysis (Michaux et al. 2007), and geometric morphometric analyses of mice collected across habitats confirmed repeated association between mandible shape differences, particularly when 25 landmarks were used. Mandible shape was correlated with δ^15^N, indicating a strong eco‐functional link between trophic ecology and morphology. While shape differences were relatively subtle and not always parallel across forests, the consistent habitat‐level clustering across sites suggests that dietary plasticity interacts with genetic drift to generate repeatable eco‐morphological divergence. This interpretation is also supported by evidence that rodent mandibles show morphological plasticity: inbred laboratory mice raised on diets of differing hardness develop shape differences affecting mechanical efficiency (Anderson et al. 2014). Thus, jaw shape variation in *A. sylvaticus* is unlikely to be directly related to genetic population structure but rather a functional consequence of habitat‐linked dietary ecology.

## 5 Conclusions

Earlier studies have speculated on the potential roles of ecological factors in shaping the population structure and evolution of *A. sylvaticus* (Montgomery and Montgomery 1989; Renaud 2005) but lacked conclusive empirical support. By integrating genetic, isotopic, and morphological data, we provide multiple independent lines of evidence that habitat mosaics promote divergence in this highly vagile generalist, with adaptation having evolved independently at multiple sites likely in response to habitat‐specific trophic ecology. Rapid evolution in mammals is often associated with insular populations, and insular rodents have demonstrated an intrinsic evolutionary potential when confronted with environmental change (Adler and Levins 1994; Hennekam et al. 2023; Renaud and Auffray 2009). Our findings suggest that such an evolutionary potential is not restricted to insular populations “sensu stricto” but also applies to those occupying mosaic landscapes, even where, geographical range, habitat range and vagility are extensive. This indicates that even widespread, common species in apparently continuous landscapes can harbor cryptic, fine‐scale population structure. Such local adaptation likely contributes to the resilience but maybe vulnerable to erosion under increasing pressures such as habitat loss, fragmentation, invasive species, climate change, and disease (Shaw et al. 2025). These threats could compromise the evolutionary adaptability of small mammals and their capacity to cope with spatially local and short‐term fluctuations in resource availability and should be considered even where species are evidently common and familiar.

## Author Contributions

**Warren Booth:** conceptualization (equal), data curation (equal), formal analysis (equal), funding acquisition (supporting), investigation (equal), methodology (equal), project administration (equal), resources (supporting), supervision (supporting), validation (equal), visualization (equal), writing – original draft (lead), writing – review and editing (equal). **W. Ian Montgomery:** conceptualization (equal), data curation (equal), funding acquisition (equal), investigation (equal), methodology (equal), project administration (equal), resources (equal), supervision (equal), writing – original draft (supporting), writing – review and editing (equal). **Lindsay S. Miles:** formal analysis (equal), investigation (supporting), methodology (supporting), software (equal), visualization (equal), writing – original draft (supporting), writing – review and editing (supporting). **Stephen H. Montgomery:** formal analysis (supporting), investigation (supporting), methodology (supporting). **Chris Harrod:** conceptualization (equal), data curation (equal), formal analysis (equal), funding acquisition (equal), investigation (equal), methodology (equal), resources (equal), software (equal), validation (equal), visualization (equal), writing – original draft (supporting), writing – review and editing (equal). **Anja Schunke:** data curation (supporting), formal analysis (equal), investigation (equal), methodology (equal), resources (equal), visualization (equal), writing – review and editing (supporting). **Paulo A. Prodöhl:** conceptualization (equal), data curation (equal), formal analysis (equal), funding acquisition (equal), investigation (equal), methodology (equal), project administration (equal), resources (equal), software (equal), supervision (equal), visualization (equal), writing – original draft (equal), writing – review and editing (equal).

## Funding

This work was supported by the Department of Agriculture and Rural Development for Northern Ireland.

## Conflicts of Interest

The authors declare no conflicts of interest.

## Supporting information


**Data S1:** ece372588‐sup‐0001‐Supinfo01.doc.

## Data Availability

Data are available from figshare: https://doi.org/10.6084/m9.figshare.28860671.

## References

[ece372588-bib-0001] Adler, G. H. , and R. Levins . 1994. “The Island Syndrome in Rodent Populations.” Quarterly Review of Biology 69: 473–490. 10.1086/418744. 7855232

[ece372588-bib-0002] Anderson, P. S. L. , S. Renaud , and E. J. Rayfield . 2014. “Adaptive Plasticity in the Mouse Mandible.” BMC Evolutionary Biology 14: 1–9. 10.1186/1471-2148-14-85. PMC4002541 24742055

[ece372588-bib-0003] Balkenhol, N. , S. Cushman , A. Stoffer , and L. Waits . 2015. Landscape Genetics: Concepts, Methods, Applications. John Wiley and Sons. 10.1002/9781118525258.

[ece372588-bib-0004] Baptista, L. , H. Meimberg , S. P. Ávila , A. M. Santos , and M. Curto . 2021. “Dispersal Ability, Habitat Characteristics, and Sea‐Surface Circulation Shape Population Structure of *Cingula trifasciata* (Gastropoda: Rissoidae) in the Remote Azores Archipelago.” BMC Ecology and Evolution 21: 128. 10.1186/s12862-021-01862-1. 34157972 PMC8218459

[ece372588-bib-0005] Barker, F. 2002. “Determination of the Population and Kin Structure of the Endemic Hosts of the Cowpox Virus, the Bank Vole, *Clethrionomys glareolus*, and the Wood Mouse, *Apodemus sylvaticus* .” PhD thesis, University of Liverpool: UK.

[ece372588-bib-0006] Berkmoes, V. , J. Scheirs , K. Jordaes , R. Blust , T. Backaljau , and R. Verhagen . 2005. “Effects of Environmental Pollution on Microsatellite DNA Diversity in Wood Mouse (*Apodemus sylvaticus*) Populations.” Environmental Toxicology and Chemistry 24: 2898–2907. 10.1897/04-483r.1. 16398127

[ece372588-bib-0007] Biello, R. , A. Brunelli , G. Sozio , et al. 2022. “The Genetic Structure and Connectivity in Two Sympatric Rodent Species With Different Life Histories Are Similarly Affected by Land Use Disturbances.” Conservation Genetics 24: 59–72. 10.1007/s10592-022-01485-z.

[ece372588-bib-0008] Boell, L. , and D. Tautz . 2011. “Micro‐Evolutionary Divergence Patterns of Mandible Shapes in Wild House Mice (*Mus musculus*) Populations.” BMC Ecology and Evolution 11: 306. 10.1186/1471-2148-11-306. PMC3213108 22008647

[ece372588-bib-0009] Booth, W. 2005. “DNA Profiling in the Wood Mouse (*Apodemus sylvaticus* L.): A Biological Model System for the Under‐Standing of Rapid Changes in Population Genetic Structure and Dynamics.” PhD thesis, Queen's University of Belfast.

[ece372588-bib-0010] Booth, W. , W. I. Montgomery , and P. A. Prodöhl . 2007. “Polyandry by Wood Mice in Natural Populations.” Journal of Zoology 273: 176–182. 10.1111/j.1469-7998.2007.00312.x.

[ece372588-bib-0011] Booth, W. , W. I. Montgomery , and P. A. Prodöhl . 2009. “Spatial Genetic Structuring in a Vagile Species, the European Wood Mouse (*Apodemus sylvaticus*).” Journal of Zoology 279: 219–228. 10.1111/j.1469-7998.2009.00608.x.

[ece372588-bib-0012] Bowler, D. E. , and T. G. Benton . 2005. “Causes and Consequences of Animal Dispersal Strategies: Relating Individual Behavior to Spatial Dynamics.” Biological Reviews 80: 205–225. 10.1017/S1464793104006645. 15921049

[ece372588-bib-0013] Brum, M. N. , N. C. Cáceres , and J. M. Bubadué . 2022. “Evolutionary Rates, Disparity, and Ecomorphology of the Mandible in American Marsupials.” Journal of Mammalian Evolution 30: 33–46. 10.1007/s10914-022-09629-1.

[ece372588-bib-0014] Bustillo‐de la Rosa, D. , A. Barrero , J. Traba , J. T. García , M. B. Morales , and E. Vásquez‐Domínguez . 2024. “Landscape Features Influencing Gene Flow and Connectivity of an Endangered Passerine.” Ecology and Evolution 14: e11078. 10.1002/ece3.11078. 38756688 PMC11097005

[ece372588-bib-0015] Cancellare, I. A. , E. M. Kiereoka , J. Janecka , B. Weckworth , R. T. Kazmaier , and R. Ward . 2021. “Multiscale Patterns of Isolation by Ecology and Fine‐Scale Population Structure in Texas Bobcats.” PeerJ 9: e11498. 10.7717/peerj.11498. 34141475 PMC8180196

[ece372588-bib-0016] Davis, J. M. , and J. A. Stamps . 2004. “The Effect of Natal Experience on Habitat Preferences.” Trends in Ecology & Evolution 19: P411–P416. 10.1016/j.tree.2004.04.006. 16701298

[ece372588-bib-0017] DeNiro, M. J. , and S. Epstein . 1981. “Influence of Diet on the Distribution of Nitrogen Isotopes in Animals.” Geochimica et Cosmochimica Acta 45: 341–351. 10.1016/0016-7037(81)90244-1.

[ece372588-bib-0018] Evanno, G. , S. Regnaut , and J. Goudet . 2005. “Detecting the Number of Clusters of Individuals Using the Software STRUCTURE: A Simulation Study.” Molecular Ecology 14: 2611–2620. 10.1111/j.1365-294X.2005.02553.x. 15969739

[ece372588-bib-0019] Feder, J. L. , S. P. Egan , and A. A. Forbes . 2012. “Ecological Adaptation and Speciation: The Evolutionary Significance of Habitat Avoidance as a Postzygotic Reproductive Barrier to Gene Flow.” International Journal of Ecology 2012: E456375. 10.1155/2012/456374.

[ece372588-bib-0020] Garcia, F. J. , M. Diaz , J. M. de Alba , et al. 1998. “Edge Effects and Patterns of Winter Abundance of Wood Mice *Apodemus sylvaticus* in Spanish Fragmented Forests.” Acta Theriologica 43: 255–262. 10.4098/at.arch.98-20.

[ece372588-bib-0021] Gockel, J. B. , C. Harr , W. Schlötterer , G. Arnold , G. Gerlach , and D. Tautz . 1997. “Isolation and Characterisation of Microsatellite Loci From *Apodemus flavicollis* (Rodentia, Muridae) and *Clethrionomy glareolus* (Rodentia, Cricetidae).” Molecular Ecology 6: 597–599. 10.1046/j.1365-294X.1997.00222.x. 9200832

[ece372588-bib-0022] Gortat, T. , A. Gryczyńska‐Siemiatkowska , R. Rutkowski , et al. 2010. “Landscape Pattern and Genetic Structure of a Yellow‐Necked Mouse *Apodemus flavicollis* Population in North‐Eastern Poland.” Acta Theriologica 55: 109–121. 10.4098/j.at.0001-7051.102.2009.

[ece372588-bib-0023] Hannebaum, S. L. , C. R. Brown , and W. Booth . 2017. “Ecological and Phenotypic Effects on Survival and Habitat Transitions of White‐Footed Mice.” Journal of Mammalogy 98: 1356–1366. 10.1093/jmammal/gyx093.

[ece372588-bib-0024] Harr, B. , K. Musolf , and G. Gerlach . 2000. “Characterisation and Isolation of DNA Microsatellite Primers in Wood Mice (*Apodemus sylvaticus*, Rodentia).” Molecular Ecology 9: 1661–1686. 10.1046/j.1365-294x.2000.01043-3.x. 11050563

[ece372588-bib-0025] Harris, S. , and D. Yalden . 2008. Mammals of the British Isles: Handbook. Mammal Society.

[ece372588-bib-0026] Haughland, D. L. , and K. W. Larsen . 2004. “Exploration Correlates With Settlement: Red Squirrel Dispersal in Contrasting Habitats.” Journal of Animal Ecology 73: 1024–1034. 10.1111/j.0021-8790.2004.00884.x.

[ece372588-bib-0027] Hennekam, J. J. , V. L. Herridge , and P. G. Cox . 2023. “Feeding Biomechanics Reveals Niche Differentiation Related to Insular Gigantism.” Evolution 77: 1303–1314. 10.1093/evolut/qpad041. 36881990

[ece372588-bib-0028] Holderegger, R. , U. Kamm , and F. Gugerli . 2006. “Adaptive Versus Neutral Genetic Diversity: Implications for Landscape Genetics.” Landscape Ecology 21: 797–807. 10.1007/s10980-005-5245-9.

[ece372588-bib-0029] Hooven, N. D. , M. T. Springer , C. K. Nielsen , and E. M. Schauber . 2023. “Influence of Natal Habitat Preference on Habitat Selection During Extra‐Home Range Movements in a Large Ungulate.” Ecology and Evolution 13: e9794. 10.1002/ece3.9794. 36760707 PMC9897958

[ece372588-bib-0030] House of Commons Report . 1971. “Northern Ireland.” Parliament, Issues 2107–2154: 253.

[ece372588-bib-0031] Johnson, O. , C. C. Ribas , A. Aleixo , L. N. Naka , M. G. Harvey , and R. T. Brumfield . 2023. “Amazonian Birds in More Dynamic Habitats Have Less Population Genetic Structure and Higher Gene Flow.” Molecular Ecology 32: 2086–2205. 10.1111/mec.16886. 36798996

[ece372588-bib-0032] Jombart, T. , and I. Ahmed . 2011. “Adegenet 1.3‐1: New Tools for the Analysis of Genome‐Wide SNP Data.” Bioinformatics 27: 3070–3071. 10.1093/bioinformatics/btr521. 21926124 PMC3198581

[ece372588-bib-0033] Jombart, T. , S. Devillard , and F. Balloux . 2010. “Discriminant Analysis of Principal Components: A New Method for the Analysis of Genetically Structured Populations.” BMC Genetics 11: 94. 10.1186/1471-2156-11-94. 20950446 PMC2973851

[ece372588-bib-0034] Kalinowski, S. T. 2005. “HP‐Rare 1.0: A Computer Program for Performing Rarefaction on Measures of Allelic Diversity.” Molecular Ecology Notes 5: 187–189. 10.1111/j.1471-8286.2004.00845.x.

[ece372588-bib-0035] Keenan, K. , P. McGinnity , T. F. Cross , W. W. Crozier , and P. A. Prodöhl . 2013. “DiveRsity: An R Package for the Estimation and Exploration of Population Genetics Parameters and Their Associated Errors.” Methods in Ecology and Evolution 4: 782–788. 10.1111/2041-210X.12067.

[ece372588-bib-0036] Klingenberg, C. P. , L. J. Leamy , and J. M. Cheverud . 2004. “Integration and Modularity of Quantitative Trait Locus Effects on Geometric Shape in the Mouse Mandible.” Genetics 166: 1909–1921. 10.1093/genetics/166.4.1909. 15126408 PMC1470826

[ece372588-bib-0037] Kopelman, N. M. , J. Mayzel , M. Jakobsson , N. A. Rosenberg , and I. Mayrose . 2015. “CLUMPAK: A Program for Identifying Clustering Modes and Packaging Population Structure Inferences Across *K* .” Molecular Ecology Resources 15: 1179–1191. 10.1111/1755-0998.12387. 25684545 PMC4534335

[ece372588-bib-0038] Kozakiewicz, M. , and E. Jurasińska . 1989. “The Role of Habitat Barriers in Woodlot Recolonization by Small Mammals.” Ecography 12: 106–111. 10.1111/j.1600-0587.1989.tb00828.x.

[ece372588-bib-0039] Leberg, P. L. 2002. “Estimating Allelic Richness: Effects of Sample Size and Bottlenecks.” Molecular Ecology 11: 2445–2449. 10.1046/j.1365-294x.2002.01612.x. 12406254

[ece372588-bib-0040] Mabry, K. E. , and J. A. Stamps . 2008. “Dispersing Brush Mice Prefer Habitat Like Home.” Proceedings of the Royal Society B: Biological Sciences 275: 1634. 10.1098/rspb.2007.1541. PMC2596818 18077253

[ece372588-bib-0041] Maestri, R. , B. D. Patterson , R. Fornel , L. R. Monteiro , and T. R. O. De Freitas . 2016. “Diet, Bite Force and Skill Morphology in the Generalist Rodent Morphotype.” Journal of Evolutionary Biology 29: 2191–2204. 10.1111/jeb.12937. 27470674

[ece372588-bib-0042] Makova, K. D. , J. C. Patton , E. Y. U. Krysanov , R. K. Chesser , and R. J. Baker . 1998. “Microsatellite Markers in Wood Mouse and Striped Field Mouse (Genus *Apodemus*).” Molecular Ecology 7: 247–255. 10.1111/j.1365-294X.1998.00315.x. 9532763

[ece372588-bib-0081] Maritinez, A. S. , J. R. Willoughby , and M. R. Christie . 2018. “Genetic Diversity in Fishes is Influenced by Habitat Type and Life‐History Variation.” Ecology and Evolution 8: 12022–12031. 30598796 10.1002/ece3.4661 PMC6303716

[ece372588-bib-0043] Martínez, J. J. , V. Millien , A. Coda , and J. Priotto . 2025. “Dietary and Habitat Use (Non)specializations Contribute to Shaping the Craniomandibular Variation and Developmental Instability in a Rodent Community.” Journal of Zoology 325: 196–209. 10.1111/jzo.13244.

[ece372588-bib-0044] Matthysen, E. 2012. “Multicausality of Dispersal: A Review.” In Dispersal Ecology and Evolution, edited by J. Clobert , M. Baguette , T. G. Benton , et al., 3–18. Oxford University Press. 10.1093/acprof:oso/9780199608898.003.0001.

[ece372588-bib-0045] McCracken, G. F. , and J. W. Bradbury . 1981. “Social Organization and Kinship in the Polygynous Bat ( *Phyllostomus hastatus* ).” Behavioral Ecology and Sociobiology 8: 11–34. 10.1007/BF00302840.

[ece372588-bib-0046] Meirmans, P. G. 2015. “Seven Common Mistakes in Population Genetics and How to Avoid Them.” Molecular Ecology 24: 3223–3231. 10.1111/mec.13243. 25974103

[ece372588-bib-0047] Michaux, J. , P. Chevret , and S. Renaud . 2007. “Morphological Diversity of Old World Rats and Mice (Rodentia, Muridae) Mandible in Relation With Phylogeny and Adaptation.” Journal of Zoological Systematics and Evolutionary Research 45: 263–279. 10.1111/j.1439-0469.2006.00390.x.

[ece372588-bib-0048] Minagawa, M. , and E. Wada . 1984. “Stepwise Enrichment of ^15^N Along Food Chains: Further Evidence and the Relationship Between d^15^N and Animal Age.” Geochimica et Cosmochimica Acta 48: 1135–1140. 10.1016/0016-7037(84)90204-7.

[ece372588-bib-0049] Montgomery, S. S. J. , and W. I. Montgomery . 1989. “Spatial and Temporal Variation in the Infracommunity Structure of Helminths of *Apodemus sylvaticus* (Rodentia: Muridae).” Parasitology 98: 145–150. 10.1017/S0031182000059783. 2717214

[ece372588-bib-0050] Montgomery, S. S. J. , and W. I. Montgomery . 1990. “Intra‐Population Variation in the Diet of the Wood Mouse *Apodemus sylvaticus* .” Journal of Zoology 222: 641–651. 10.1111/j.1469-7998.1990.tb06020.x.

[ece372588-bib-0051] Montgomery, W. I. , and J. Gurnell . 1985. “The Behaviour of *Apodemus* .” Symposium of the Zoological Society of London 55: 89–115.

[ece372588-bib-0052] Nosil, P. , S. P. Egan , and D. J. Funk . 2008. “Heterogeneous Genomic Differentiation Between Walking‐Stick Ecotypes: “Isolation by Adaptation” and Multiple Roles for Divergent Selection.” Evolution 62: 316–336. 10.1111/j.1558-5646.2007.00299.x. 17999721

[ece372588-bib-0053] Nosil, P. , T. H. Vines , and D. J. Funk . 2007. “Reproductive Isolation Caused by Natural Selection Against Immigrants From Divergent Habitats.” Evolution 59: 705–719. 10.1111/j.0014-3820.2005.tb01747.x. 15926683

[ece372588-bib-0054] O'Neill, K. P. 2001. “Dispersal and Spatial Ecology in Woodmice Living in Pastoral Farmland.” PhD thesis, Queen's University of Belfast.

[ece372588-bib-0055] Orgeret, F. , U. G. Kormann , B. Catitti , et al. 2024. “Imprinted Habitat Selection Varies Across Dispersal Phases in a Raptor Species.” Scientific Reports 14: 26656. 10.1038/s41598-024-75815-1. 39496617 PMC11535207

[ece372588-bib-0056] Piper, W. H. , M. W. Palmer , N. Banfield , and M. W. Meyer . 2013. “Can Settlement in Natal‐Like Habitat Explain Maladaptive Habitat Selection.” Proceedings of the Royal Society B: Biological Sciences 280: 1765. 10.1098/rspb.2013.0979. PMC3712445 23804619

[ece372588-bib-0057] Porlier, M. , D. Garant , P. Perret , and A. Charmantier . 2012. “Habitat‐Linked Population Genetic Differentiation in the Blue Tit *Cyanoses caeruleus* .” Journal of Heredity 103: 781–791. 10.1093/jhered/ess064. 23087385

[ece372588-bib-0058] Pritchard, J. K. , M. Stephens , and P. Donnelly . 2000. “Inference of Population Structure Using Multilocus Genotype Data.” Genetics 155: 945–959. 10.1093/genetics/155.2.945. 10835412 PMC1461096

[ece372588-bib-0059] Ravinet, M. , P. A. Prodöhl , and C. Harrod . 2013. “Parallel and Nonparallel Ecological, Morphological and Genetic Divergence in Lake‐Stream Stickleback From a Single Catchment.” Journal of Evolutionary Biology 26: 186–204. 10.1111/jeb.12049. 23199201

[ece372588-bib-0060] Renaud, S. 2005. “First Upper Molar and Mandible Shape of Wood Mice ( *Apodemus sylvaticus* ) From Northern Germany: Ageing, Habitat and Insularity.” Mammalian Biology – Zeitschrift Fur Saugetierkunde 70: 157–170. 10.1016/j.mambio.2004.10.004.

[ece372588-bib-0061] Renaud, S. , and J.‐C. Auffray . 2009. “Adaptation and Plasticity in Insular Evolution of the House Mouse Mandible.” Journal of Zoological Systematics and Evolutionary Research 48: 138–150. 10.1111/j.1439-0469.2009.00527.x.

[ece372588-bib-0062] Renaud, S. , and J. R. Michaux . 2003. “Adaptive Latitudinal Trends in the Mandible Shape of *Apodemus* Mice.” Journal of Biogeography 30: 1617–1628. 10.1046/j.1365-2699.2003.00932.x.

[ece372588-bib-0063] Rogers, L. M. , and M. I. Gorman . 1995. “The Diet of the Wood Mouse *Apodemus sylvaticus* on Set‐Aside Land.” Journal of Zoology 235: 77–83. 10.1111/j.1469-7998.1995.tb05129.x.

[ece372588-bib-0064] Rohlf, F. J. 2006. “tpsDig.” State University of New York at Stony Brook, New York.

[ece372588-bib-0065] Rudyk, A. I. , K. Kuprina , A. M. Bergaliev , et al. 2025. “Genetic Diversity and Population Structure of the Subterranean Rodent, Northern Mole Vole ( *Ellobius talpinus* ).” Mammalian Biology 105: 571–588. 10.1007/s42991-025-00498-8.

[ece372588-bib-0066] Samuels, J. X. 2009. “Cranial Morphology and Dietary Habits of Rodents.” Zoological Journal of the Linnean Society 156: 864–888. 10.1111/j.1096-3642.2009.00502.x.

[ece372588-bib-0067] Schroeder, L. , S. Elton , and R. R. Ackermann . 2022. “Skull Variation in Afro‐Eurasian Monkeys Results From Both Adaptive and Non‐Adaptive Evolutionary Processes.” Scientific Reports 12: 12516. 10.1038/s41598-022-16734-x. 35869137 PMC9307787

[ece372588-bib-0068] Selonen, V. , I. K. Hanski , and A. Desrochers . 2007. “Natal Habitat‐Biased Dispersal in the Siberian Flying Squirrel.” Proceedings of the Royal Society B 274: 2063–2068. 10.1098/rspb.2007.0570. 17567559 PMC2275184

[ece372588-bib-0069] Shaw, R. E. , K. A. Farquharson , M. W. Bruford , et al. 2025. “Global Meta‐Analysis Shows Action Is Needed to Halt Genetic Diversity Loss.” Nature 638: 704–710. 10.1038/s41586-024-08458-x. 39880948 PMC11839457

[ece372588-bib-0070] Sheets, H. D. 2003. IMP Software Series. Canisius College.

[ece372588-bib-0071] Singleton, G. R. , and D. A. Hay . 1983. “The Effect of Social Organization on Reproductive Success and Gene Flow in Colonies of Wild House Mice, *Mus musculus* .” Behavioral Ecology and Sociobiology 12: 49–56. 10.1007/BF00296932.

[ece372588-bib-0072] Stamps, J. A. , and R. R. Swaisgood . 2007. “Someplace Like Home: Experience, Habitat Selection and Conservation Biology.” Applied Animal Behaviour Science 102: 392–409. 10.1016/j.applanim.2006.05.038.

[ece372588-bib-0073] Szulkin, M. , P.‐A. Gagnaire , N. Bierne , and A. Charmantier . 2016. “Population Genomic Footprints of Fine‐Scale Differentiation Between Habitats in Mediterranean Blue Tits.” Molecular Ecology 25: 542–558. 10.1111/mec.13486. 26800038

[ece372588-bib-0074] Taggart, J. B. , R. A. Hynes , P. A. Prodöhl , and A. Ferguson . 1992. “A Simplified Protocol for Routine Total DNA Isolation From Salmonid Fishes.” Journal of Fish Biology 40: 963–965. 10.1111/j.1095-8649.1992.tb02641.x.

[ece372588-bib-0075] Tattersall, F. H. , D. W. Macdonald , B. J. Hart , and W. Manley . 2004. “Balanced Dispersal or Source–Sink–Do Both Models Describe Wood Mice in Farmed Landscapes.” Oikos 106: 536–550. 10.1111/j.0030-1299.2004.13114.x.

[ece372588-bib-0082] Telleria, J. L. , T. Santos , and M. Alcantara . 1991. “Abundance and Food‐Searching Intensity of Wood Mice (*Apodemus sylvaticus*) in Fragmented Forests.” Journal of Mammalogy 72: 183–187.

[ece372588-bib-0076] von Cramon‐Taubadel, N. 2011. “Global Human Mandibular Variation Reflects Differences in Agricultural and Hunter‐Gather Subsistence Strategies.” Proceedings of the National Academy of Sciences of the United States of America 108: 19546–19551. 10.1073/pnas.1113050108. 22106280 PMC3241821

[ece372588-bib-0077] Weir, B. S. , and C. C. Cockerham . 1984. “Estimating F‐Statistics for the Analysis of Population Structure.” Evolution 38: 1358–1370. 10.2307/2408641. 28563791

[ece372588-bib-0078] Wilson, A. , B. Fenton , G. Malloch , B. Boag , S. Hubbard , and G. Begg . 2016. “Urbanization Versus Agriculture: A Comparison of Local Genetic Diversity and Gene Flow Between Wood Mouse *Apodemus sylvaticus* Populations in Human‐Modified Landscapes.” Ecography 39: 89–97. 10.1111/ecog.01297.

[ece372588-bib-0079] Wolton, R. 1985. “The Ranging and Nesting Behavior of Wood Mice, *Apodemus sylvaticus* (Rodentia: Muridae), as Revealed by Radio‐Tracking.” Journal of Zoology 206: 203–224. 10.1111/j.1469-7998.1985.tb05645.x.

[ece372588-bib-0080] Wolton, R. J. , and J. R. Flowerdew . 1985. “Spatial Distribution and Movements of Wood Mice, Yellow‐Necked Mice and Bank Voles.” Symposium of the Zoological Society of London 55: 249–275. 10.1134/S106741360907011X.

