# Supplementary material for: Habitat Mosaic Limits Gene Flow and Promotes Morphological Adaptation in a Generalist Mammal
Source: Ecol Evol. 2025 Dec 3;15(12):e72588. doi: 10.1002/ece3.72588 (PMC12675938; doi:10.1002/ece3.72588)

Supplemental Material: Habitat mosaic limits gene flow and promotes morphological adaptation in a generalist mammal

Warren Booth^1*^, W. Ian Montgomery^2^, Lindsay S. Miles^1^, Stephen H. Montgomery^3^, Chris Harrod^4^, Anja Schunke^5^ and Paulo A. Prodöhl^2^

ORCID

WB – 0000-0003-2355-0702; WIM – 0000-0001-9715-4767; LSM – 0000-0003-0406-7985; SHM – 000-0002-5474-5695; CH – 0000-0002-5353-1556; PAP – 0000-0001-8570-9964

^1^ Department of Entomology, Virginia Polytechnic Institute and State University, Blacksburg, VA, USA

^2^ School of Biological Sciences, Queen’s University Belfast, Belfast, Northern Ireland, UK

^3^ School of Biological Sciences, University of Bristol, Bristol, United Kingdom, UK

^4^ Scottish Centre for Ecology and the Natural Environment, School of Biodiversity, One Health and Veterinary Medicine, University of Glasgow, Glasgow, UK

^5^ DogPersonality, Plön, Germany

*To whom correspondence should be addressed. E-mail: [warrenbooth@vt.edu](mailto:warrenbooth@vt.edu)

**Supplemental Methods**

Mitochondrial DNA PCR-RFLP analysis was carried out on a subset of samples collected from Tollymore Forest Park during 2005. Species-specific primers WMP2F (5’-ATC ACT TGT TCC TTA ATT AGG GAC TA-3’) and WMP2R (5'-AGA TTG AAG CCA GTA AGT AGG GTA TT-3'), were used to amplify an ~3.14 kilobase (kb) fragment spanning parts of the 16S rRNA and tRNA-ASN genes. PCRs for RFLP analysis consisted of ~100 ng template DNA, 10 pmol of each primer, 200 μM dNTP mix, 2.5 mM MgCl_2_, 1× *Taq* polymerase buffer and 2 U of *Taq* polymerase (Invitrogen). Amplifications were performed in 50 μl volumes with a 5 min denaturation at 94^o^C, 30 cycles of 94^o^C for 1 min, 58^o^C for 1 min and 72^o^C for 1 min, followed by a final extension at 72^o^C for 10 min. Restriction digests with *Alu*I and *Hae*III were carried out in 12 μl volumes using 8 μl of PCR product, 3 U of restriction enzymes and the appropriate buffer and conditions as recommended by the manufacturer (NEB/Promega). Restriction fragments were separated by electrophoresis in Tris–Borate–EDTA buffered agarose gels containing ethidium bromide. Resultant mtDNA fragment profiles were visualized using UV light. The fragment sizes of each restriction morph were calculated by comparing migration distances relative to 1 kb and 100 bp DNA Ladders (Invitrogen). MtDNA restriction fragment data was analysed using Arlequin 3.1 (43).

**Supplemental Results**

The screening of the 3.14 kb mtDNA region for the two restriction enzymes revealed three composite haplotypes among samples. In most instances, a clear partitioning of mtDNA haplotype frequency distribution was observed among different habitat types (Fig S3). No differences were observed between sexes.

**Supplemental References**

Goudet, J., N. Perrin, and P. Waser. 2002. “Tests for sex-biased dispersal using bi-parentally inherited genetic markers.” *Molecular Ecology* 11: 1103-1114.

Laurent, E., G. Laval, and S. Schneider. 2005. “Arlequin (version 3.0): an integrated software package for population genetics data analysis.” *Evolutionary Bioinformatics Online* 1: 47-50. <https://doi.org/10.1177/117693430500100003>.

**Table S1.** Pairwise *F*_ST_ values (above diagonal) and their 95% confidence intervals (below diagonal) for wood mice (*Apodemus sylvaticus*) sampled in Northern Ireland. Abbreviations denote forest park, habitat type, and sampling year: T = Tollymore, C = Castlewellan, R = Rostrevor; H = hedgerow, E = forest edge, I = inner forest; 04 = 2004, 05 = 2005. For example, TH04 = Tollymore hedgerow sampled in 2004.

**Table S2**. Number of wood mouse, *Apodemus sylvaticus*, specimens used in the morphometric analyses. NB: there was no significant effect of sex on mandible shape.

|  |  | Habitat | | |
| --- | --- | --- | --- | --- |
| Site | Sex | Hedgerow | Forest edge | Inner-forest |
| Tollymore | Male | 4 | 10 | 7 |
|  | Female | 3 | 8 | 9 |
| Castlewellan | Male | 3 | 4 | 3 |
|  | Female | 5 | 1 | 3 |
| Rostrevor | Male | 6 | 5 | 3 |
|  | Female | 3 | 6 | 4 |

**Figure S1.** Virtual radiograph of left hemimandible showing position of landmarks, blue: 15 standard landmarks, red: 10 additional landmarks (see text for details).


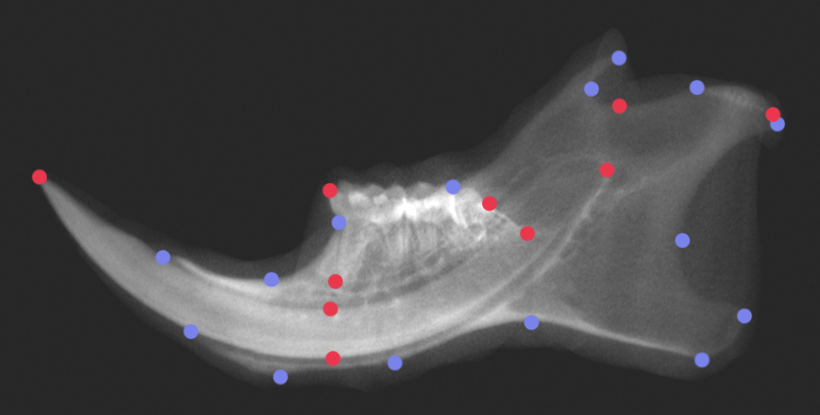


**Figure S2.** Summary of Bayesian clustering analysis (STRUCTURE) for wood mice (*Apodemus sylvaticus*) sampled across 2004 and 2005 from Tollymore Forest park (T) in Northern Ireland. Individuals were collected from hedgerows (H), forest edges (E), and inner forests (I) Each vertical bar represents one individual, partitioned into genetic clusters according to estimated membership coefficients. Analyses were performed across K = 2–9, with results summarized using CLUMPAK. The figure illustrates clear subdivision among habitat types but temporal stability among pair-matched sites across years.


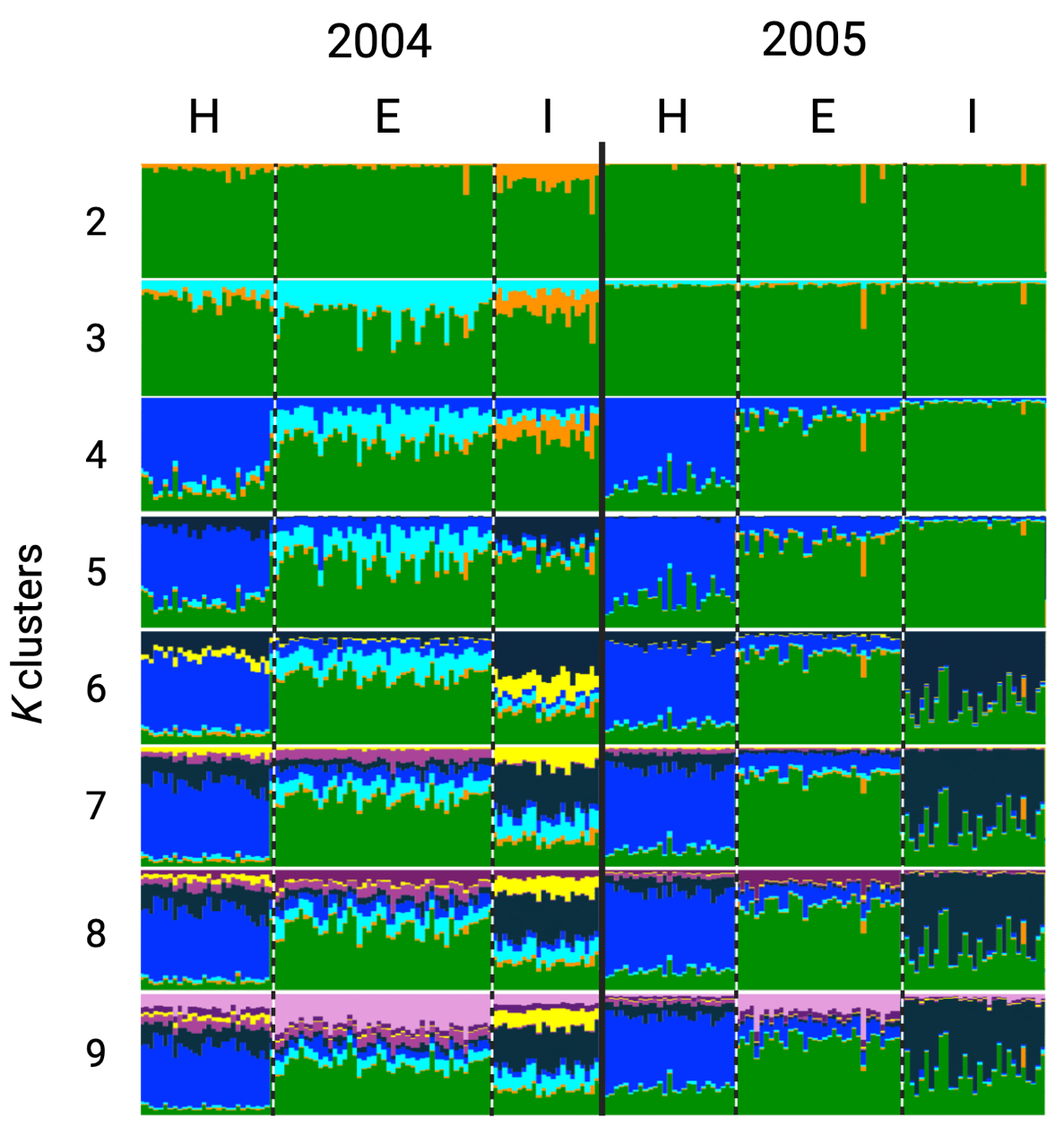


**Figure S3.** Sex-bias dispersal, test from [Goudet et al (2002)](https://doi.org/10.1046/j.1365-), shows no significant bias (t = 1.522919, *p* = 0.137) between males and females across all populations combined. AI_C_ – Corrected Assignment Index.


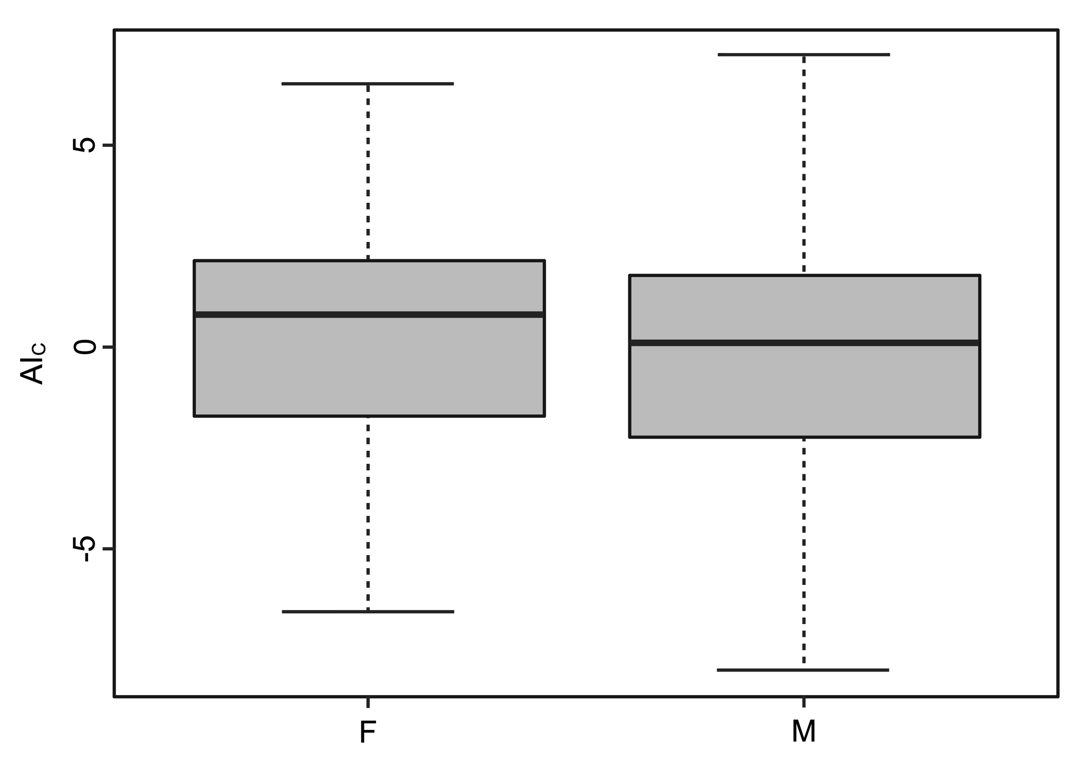


**Figure S4.** Frequency distribution of composite 16S mtDNA haplotypes among *A. sylvaticus* sampled across habitat types for the Tollymore.


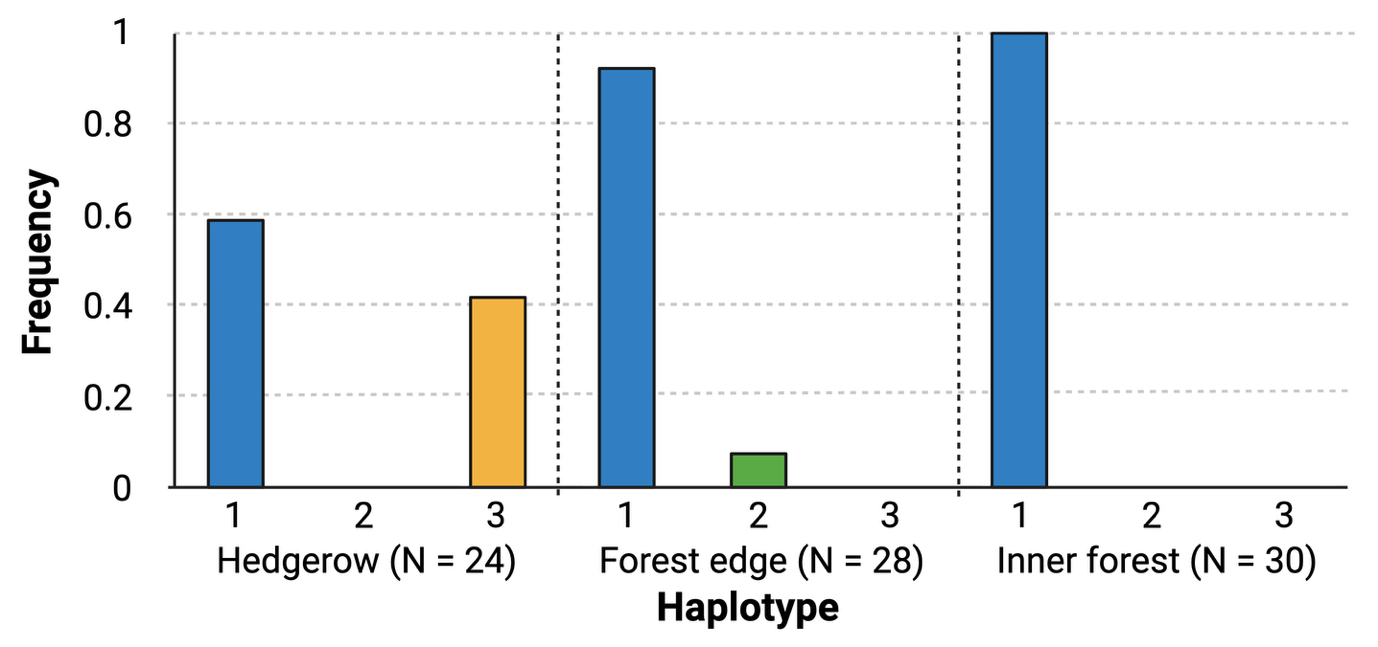

Supplement: Supplementary file 1 — Data S1: ece372588‐sup‐0001‐Supinfo01.doc. [file ECE3-15-e72588-s001.doc]
